# Dichloridobis[4-(1*H*-pyrazol-3-yl)pyridine-κ*N* ^1^]zinc

**DOI:** 10.1107/S1600536811037585

**Published:** 2011-09-30

**Authors:** Zheng-De Tan, Feng-Jiao Tan, Bo Tan, Cheng-Ming Zhang

**Affiliations:** a College of Chemistry and Chemical Engineering, Hunan Institute of Engineering, Xiangtan 411104, People’s Republic of China; b The People’s Hospital of Xiangtan County, Xiangtan 411104, People’s Republic of China

## Abstract

In the title compound, [ZnCl_2_(C_8_H_7_N_3_)_2_], the Zn^II^ cation is coordinated by two Cl^−^ anions and two 4-(1*H*-pyrazol-3-yl)pyridine ligands in a distorted tetra­hedral geometry. In the two 4-(1*H*-pyrazol-3-yl)pyridine ligands, the dihedral angles between the pyrazole and pyridine rings are 3.3 (3) and 13.3 (3)°. Inter­molecular N—H⋯N and N—H⋯Cl hydrogen bonding is present in the crystal structure.

## Related literature

For the synthesis of 4-(1*H*-pyrazol-3-yl)-pyridine, see: Davies *et al.* (2003 ▶). For a related complex, see: Davies *et al.* (2005 ▶).
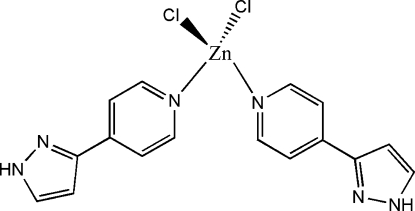


## Experimental

### 

#### Crystal data

[ZnCl_2_(C_8_H_7_N_3_)_2_]
*M* *_r_* = 426.60
Monoclinic, 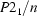

*a* = 12.306 (3) Å
*b* = 7.8827 (16) Å
*c* = 18.883 (4) Å
β = 94.82 (3)°
*V* = 1825.3 (6) Å^3^
*Z* = 4
Mo *K*α radiation
μ = 1.65 mm^−1^
*T* = 293 K
0.24 × 0.21 × 0.02 mm

#### Data collection

Rigaku SCXmini diffractometer
Absorption correction: multi-scan (*ABSCOR*; Higashi, 1995 ▶) *T*_min_ = 0.693, *T*_max_ = 0.971
14854 measured reflections
3283 independent reflections
2052 reflections with *I* > 2σ(*I*)
*R*_int_ = 0.122

#### Refinement

*R*[*F* ^2^ > 2σ(*F* ^2^)] = 0.080
*wR*(*F* ^2^) = 0.138
*S* = 1.11
3283 reflections
226 parameters
H-atom parameters constrained
Δρ_max_ = 0.42 e Å^−3^
Δρ_min_ = −0.30 e Å^−3^

### 

Data collection: *PROCESS-AUTO* (Rigaku, 2006 ▶); cell refinement: *PROCESS-AUTO*; data reduction: *PROCESS-AUTO*; program(s) used to solve structure: *SHELXS97* (Sheldrick, 2008 ▶); program(s) used to refine structure: *SHELXL97* (Sheldrick, 2008 ▶); molecular graphics: *ORTEPII* (Johnson, 1976 ▶); software used to prepare material for publication: *SHELXL97*.

## Supplementary Material

Crystal structure: contains datablock(s) I, global. DOI: 10.1107/S1600536811037585/xu5325sup1.cif

Structure factors: contains datablock(s) I. DOI: 10.1107/S1600536811037585/xu5325Isup2.hkl

Additional supplementary materials:  crystallographic information; 3D view; checkCIF report

## Figures and Tables

**Table 1:** Selected bond lengths (Å)

Zn1—N1	2.041 (4)
Zn1—N2	2.032 (4)
Zn1—Cl1	2.2395 (17)
Zn1—Cl2	2.2241 (18)

**Table 2:** Hydrogen-bond geometry (Å, °)

*D*—H⋯*A*	*D*—H	H⋯*A*	*D*⋯*A*	*D*—H⋯*A*
N4—H4*A*⋯N5^i^	0.86	2.23	2.945 (8)	140
N6—H6⋯Cl1^ii^	0.86	2.46	3.266 (5)	156

Symmetry codes: (i) 
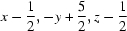
; (ii) 
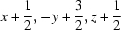
.

**Fig. 1.**
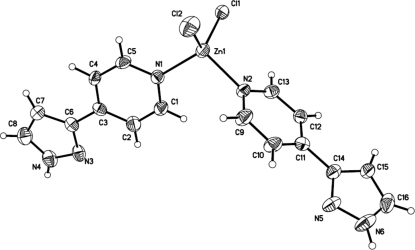
The structure of the title compound, showing the atomic numbering scheme with 30% probability displacement ellipsoids.

**Fig. 2.**
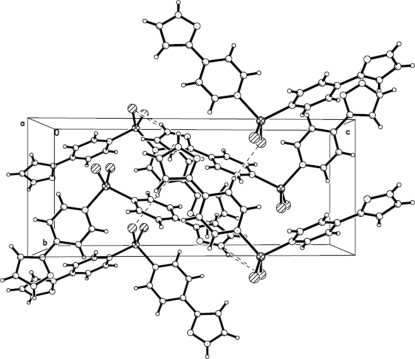
A view of the three-dimensional network. Hydrogen bonds are shown as dashed lines.

## supplementary crystallographic information

### Comment

Pyridine derivatives are an important class of ligand for constructing metal–organic frameworks. From the structural point of view, 4-(1*H*-pyrazol-3-yl)-pyridine can be used as pyridines ligand in building coordination compounds. In the present paper, we present the structure of the complex ZnCl_2_(C_8_H_7_N_3_)_2_.

As shown in Fig. 1, the Zn^II^ atom exhibits a tetrahedral coordination sphere, defined by two Cl atoms and two N atoms from two different 4-(1*H*-pyrazol-3-yl)-pyridine ligands. Intermolecular N—H···N and N—H···Cl hydrogen bonds can be seen in the three-dimensional supramolecular network of the compound (Fig. 2).

### Experimental

4-(1*H*-Pyrazol-3-yl)-pyridine was prepared according to the published method of Davies *et al.* (2003). The aqueous solution (20 ml) containing ZnCl_2_(0.1 mmol, 14 mg) and 4-(1*H*-pyrazol-3-yl)-pyridine (0.2 mmol, 29 mg) was stirred for a few minutes in air, and left to stand at room temperature for a few weeks, then the colorless crystals were obtained.

### Refinement

Carbon and nitrogen bound H atoms were placed at calculated positions and were treated as riding on the parent C or N atoms with C—H = 0.93 Å, N—H = 0.86 Å, and with *U*_iso_(H) = 1.2*U*_eq_(C,N).

### Crystal data

**Table:**

[ZnCl_2_(C_8_H_7_N_3_)_2_]	*F*(000) = 864
*M**_r_* = 426.60	*D*_x_ = 1.552 Mg m^−^^3^
Monoclinic, *P*2_1_/*n*	Mo *K*α radiation, λ = 0.71073 Å
Hall symbol: -P 2yn	Cell parameters from 13142 reflections
*a* = 12.306 (3) Å	θ = 3.1–27.7°
*b* = 7.8827 (16) Å	µ = 1.65 mm^−^^1^
*c* = 18.883 (4) Å	*T* = 293 K
β = 94.82 (3)°	Platelet, colourless
*V* = 1825.3 (6) Å^3^	0.24 × 0.21 × 0.02 mm
*Z* = 4	

### Data collection

**Table:**

Rigaku SCXmini diffractometer	3283 independent reflections
Radiation source: fine-focus sealed tube	2052 reflections with *I* > 2σ(*I*)
graphite	*R*_int_ = 0.122
ω scans	θ_max_ = 25.2°, θ_min_ = 3.1°
Absorption correction: multi-scan (*ABSCOR*; Higashi, 1995)	*h* = −14→14
*T*_min_ = 0.693, *T*_max_ = 0.971	*k* = −9→9
14854 measured reflections	*l* = −22→22

### Refinement

**Table:**

Refinement on *F*^2^	Primary atom site location: structure-invariant direct methods
Least-squares matrix: full	Secondary atom site location: difference Fourier map
*R*[*F*^2^ > 2σ(*F*^2^)] = 0.080	Hydrogen site location: inferred from neighbouring sites
*wR*(*F*^2^) = 0.138	H-atom parameters constrained
*S* = 1.11	*w* = 1/[σ^2^(*F*_o_^2^) + (0.0451*P*)^2^] where *P* = (*F*_o_^2^ + 2*F*_c_^2^)/3
3283 reflections	(Δ/σ)_max_ < 0.001
226 parameters	Δρ_max_ = 0.42 e Å^−^^3^
0 restraints	Δρ_min_ = −0.30 e Å^−^^3^

### Special details

**Table:**

Geometry. All e.s.d.'s (except the e.s.d. in the dihedral angle between two l.s. planes) are estimated using the full covariance matrix. The cell e.s.d.'s are taken into account individually in the estimation of e.s.d.'s in distances, angles and torsion angles; correlations between e.s.d.'s in cell parameters are only used when they are defined by crystal symmetry. An approximate (isotropic) treatment of cell e.s.d.'s is used for estimating e.s.d.'s involving l.s. planes.
Refinement. Refinement of *F*^2^ against ALL reflections. The weighted *R*-factor *wR* and goodness of fit *S* are based on *F*^2^, conventional *R*-factors *R* are based on *F*, with *F* set to zero for negative *F*^2^. The threshold expression of *F*^2^ > σ(*F*^2^) is used only for calculating *R*-factors(gt) *etc*. and is not relevant to the choice of reflections for refinement. *R*-factors based on *F*^2^ are statistically about twice as large as those based on *F*, and *R*-factors based on ALL data will be even larger.

### Fractional atomic coordinates and isotropic or equivalent isotropic displacement parameters (Å^2^)

**Table:**

	*x*	*y*	*z*	*U*_iso_*/*U*_eq_	
Zn1	0.46511 (6)	0.48942 (8)	0.20777 (3)	0.0476 (3)	
Cl1	0.31124 (13)	0.34083 (19)	0.21462 (8)	0.0543 (5)	
Cl2	0.61580 (14)	0.3545 (2)	0.18253 (9)	0.0761 (6)	
N3	0.3040 (4)	1.2186 (7)	0.0109 (3)	0.0555 (14)	
C5	0.4365 (5)	0.6564 (7)	0.0659 (3)	0.0502 (16)	
H5	0.4609	0.5527	0.0499	0.060*	
N1	0.4321 (4)	0.6807 (6)	0.1363 (2)	0.0435 (12)	
C3	0.3709 (4)	0.9364 (7)	0.0374 (3)	0.0421 (15)	
C6	0.3357 (4)	1.0691 (8)	−0.0137 (3)	0.0454 (15)	
C4	0.4062 (4)	0.7799 (7)	0.0171 (3)	0.0448 (16)	
H4	0.4096	0.7571	−0.0310	0.054*	
C2	0.3689 (5)	0.9619 (8)	0.1100 (3)	0.0578 (18)	
H2	0.3472	1.0663	0.1269	0.069*	
C8	0.2886 (6)	1.2202 (10)	−0.1081 (4)	0.072 (2)	
H8	0.2739	1.2588	−0.1544	0.087*	
N4	0.2750 (4)	1.3070 (7)	−0.0484 (3)	0.0653 (16)	
H4A	0.2502	1.4090	−0.0482	0.078*	
C1	0.3987 (5)	0.8344 (8)	0.1565 (3)	0.0545 (17)	
H1	0.3957	0.8548	0.2048	0.065*	
C7	0.3281 (5)	1.0644 (8)	−0.0878 (3)	0.0590 (18)	
H7	0.3459	0.9751	−0.1169	0.071*	
C14	0.5765 (5)	0.8133 (7)	0.5098 (3)	0.0460 (16)	
N6	0.6657 (5)	0.9532 (7)	0.5901 (3)	0.0718 (18)	
H6	0.7140	1.0182	0.6115	0.086*	
N5	0.6636 (4)	0.9156 (7)	0.5209 (3)	0.0592 (15)	
C16	0.5854 (7)	0.8797 (9)	0.6228 (4)	0.071 (2)	
H16	0.5727	0.8896	0.6705	0.086*	
C15	0.5263 (6)	0.7882 (8)	0.5724 (3)	0.0578 (18)	
H15	0.4650	0.7224	0.5784	0.069*	
N2	0.4966 (4)	0.6034 (6)	0.3038 (2)	0.0433 (12)	
C12	0.4504 (5)	0.6635 (7)	0.4214 (3)	0.0456 (15)	
H12	0.3991	0.6554	0.4547	0.055*	
C11	0.5491 (5)	0.7416 (7)	0.4388 (3)	0.0414 (15)	
C13	0.4283 (5)	0.5978 (7)	0.3545 (3)	0.0463 (15)	
H13	0.3610	0.5459	0.3440	0.056*	
C9	0.5921 (5)	0.6814 (8)	0.3215 (3)	0.0617 (19)	
H9	0.6419	0.6886	0.2873	0.074*	
C10	0.6208 (5)	0.7512 (8)	0.3870 (3)	0.0574 (18)	
H10	0.6880	0.8043	0.3963	0.069*	

### Atomic displacement parameters (Å^2^)

**Table:**

	*U*^11^	*U*^22^	*U*^33^	*U*^12^	*U*^13^	*U*^23^
Zn1	0.0574 (5)	0.0463 (5)	0.0385 (4)	0.0042 (4)	0.0010 (3)	−0.0037 (4)
Cl1	0.0668 (11)	0.0519 (10)	0.0422 (9)	−0.0083 (8)	−0.0073 (8)	0.0009 (8)
Cl2	0.0715 (14)	0.0823 (13)	0.0752 (13)	0.0279 (10)	0.0113 (10)	−0.0140 (11)
N3	0.055 (4)	0.059 (4)	0.053 (3)	0.013 (3)	0.007 (3)	0.018 (3)
C5	0.060 (4)	0.041 (4)	0.050 (4)	0.009 (3)	0.008 (3)	−0.012 (3)
N1	0.046 (3)	0.043 (3)	0.042 (3)	0.003 (2)	0.008 (2)	0.000 (2)
C3	0.030 (4)	0.041 (4)	0.056 (4)	−0.005 (3)	0.010 (3)	0.003 (3)
C6	0.034 (4)	0.048 (4)	0.055 (4)	0.002 (3)	0.007 (3)	0.005 (3)
C4	0.054 (4)	0.051 (4)	0.029 (3)	0.001 (3)	−0.003 (3)	0.000 (3)
C2	0.070 (5)	0.047 (4)	0.058 (4)	0.014 (3)	0.018 (4)	0.004 (4)
C8	0.078 (6)	0.078 (6)	0.060 (5)	0.004 (4)	0.000 (4)	0.013 (5)
N4	0.070 (4)	0.057 (4)	0.070 (4)	0.016 (3)	0.007 (3)	0.019 (3)
C1	0.068 (5)	0.051 (4)	0.045 (4)	0.007 (4)	0.013 (3)	−0.005 (4)
C7	0.071 (5)	0.056 (5)	0.050 (4)	−0.006 (4)	0.006 (4)	−0.005 (4)
C14	0.049 (4)	0.042 (4)	0.044 (4)	0.014 (3)	−0.011 (3)	−0.004 (3)
N6	0.066 (4)	0.068 (4)	0.075 (5)	0.022 (3)	−0.032 (3)	−0.032 (4)
N5	0.058 (4)	0.061 (4)	0.056 (4)	−0.002 (3)	−0.011 (3)	−0.021 (3)
C16	0.106 (7)	0.066 (5)	0.042 (4)	0.030 (5)	0.005 (5)	−0.003 (4)
C15	0.082 (5)	0.046 (4)	0.044 (4)	0.003 (4)	−0.003 (4)	−0.005 (3)
N2	0.046 (3)	0.045 (3)	0.038 (3)	−0.004 (3)	−0.004 (2)	−0.005 (2)
C12	0.051 (4)	0.043 (4)	0.045 (4)	−0.001 (3)	0.012 (3)	−0.001 (3)
C11	0.033 (4)	0.038 (4)	0.051 (4)	0.002 (3)	−0.005 (3)	0.003 (3)
C13	0.039 (4)	0.048 (4)	0.051 (4)	−0.003 (3)	0.002 (3)	−0.007 (3)
C9	0.059 (5)	0.074 (5)	0.054 (4)	−0.011 (4)	0.015 (4)	−0.020 (4)
C10	0.042 (4)	0.068 (5)	0.061 (5)	−0.014 (3)	−0.001 (4)	−0.016 (4)

### Geometric parameters (Å, °)

**Table:**

Zn1—N1	2.041 (4)	C1—H1	0.9300
Zn1—N2	2.032 (4)	C7—H7	0.9300
Zn1—Cl1	2.2395 (17)	C14—N5	1.344 (7)
Zn1—Cl2	2.2241 (18)	C14—C15	1.394 (8)
N3—C6	1.337 (7)	C14—C11	1.468 (7)
N3—N4	1.342 (6)	N6—N5	1.337 (6)
C5—N1	1.349 (6)	N6—C16	1.341 (8)
C5—C4	1.370 (7)	N6—H6	0.8600
C5—H5	0.9300	C16—C15	1.357 (8)
N1—C1	1.345 (7)	C16—H16	0.9300
C3—C4	1.374 (7)	C15—H15	0.9300
C3—C2	1.387 (8)	N2—C13	1.326 (6)
C3—C6	1.464 (7)	N2—C9	1.344 (7)
C6—C7	1.395 (8)	C12—C13	1.371 (7)
C4—H4	0.9300	C12—C11	1.376 (7)
C2—C1	1.366 (7)	C12—H12	0.9300
C2—H2	0.9300	C11—C10	1.372 (8)
C8—N4	1.341 (8)	C13—H13	0.9300
C8—C7	1.363 (8)	C9—C10	1.372 (8)
C8—H8	0.9300	C9—H9	0.9300
N4—H4A	0.8600	C10—H10	0.9300
			
N2—Zn1—N1	106.01 (19)	C8—C7—C6	104.5 (6)
N2—Zn1—Cl2	107.64 (15)	C8—C7—H7	127.8
N1—Zn1—Cl2	109.55 (14)	C6—C7—H7	127.8
N2—Zn1—Cl1	106.16 (15)	N5—C14—C15	110.9 (6)
N1—Zn1—Cl1	107.60 (13)	N5—C14—C11	119.6 (6)
Cl2—Zn1—Cl1	119.11 (7)	C15—C14—C11	129.5 (6)
C6—N3—N4	103.4 (5)	N5—N6—C16	113.6 (6)
N1—C5—C4	122.1 (5)	N5—N6—H6	123.2
N1—C5—H5	119.0	C16—N6—H6	123.2
C4—C5—H5	119.0	N6—N5—C14	103.6 (5)
C1—N1—C5	116.5 (5)	N6—C16—C15	106.2 (6)
C1—N1—Zn1	121.7 (4)	N6—C16—H16	126.9
C5—N1—Zn1	121.7 (4)	C15—C16—H16	126.9
C4—C3—C2	116.0 (5)	C16—C15—C14	105.7 (6)
C4—C3—C6	122.7 (6)	C16—C15—H15	127.2
C2—C3—C6	121.2 (6)	C14—C15—H15	127.2
N3—C6—C7	112.0 (5)	C13—N2—C9	115.4 (5)
N3—C6—C3	118.7 (6)	C13—N2—Zn1	123.0 (4)
C7—C6—C3	129.3 (6)	C9—N2—Zn1	121.5 (4)
C5—C4—C3	121.6 (5)	C13—C12—C11	119.4 (6)
C5—C4—H4	119.2	C13—C12—H12	120.3
C3—C4—H4	119.2	C11—C12—H12	120.3
C1—C2—C3	120.2 (6)	C10—C11—C12	117.6 (6)
C1—C2—H2	119.9	C10—C11—C14	121.1 (6)
C3—C2—H2	119.9	C12—C11—C14	121.4 (6)
N4—C8—C7	106.9 (6)	N2—C13—C12	124.3 (6)
N4—C8—H8	126.5	N2—C13—H13	117.8
C7—C8—H8	126.5	C12—C13—H13	117.8
C8—N4—N3	113.2 (5)	N2—C9—C10	124.2 (6)
C8—N4—H4A	123.4	N2—C9—H9	117.9
N3—N4—H4A	123.4	C10—C9—H9	117.9
N1—C1—C2	123.5 (6)	C9—C10—C11	119.1 (6)
N1—C1—H1	118.2	C9—C10—H10	120.4
C2—C1—H1	118.2	C11—C10—H10	120.4

### Hydrogen-bond geometry (Å, °)

**Table:**

*D*—H···*A*	*D*—H	H···*A*	*D*···*A*	*D*—H···*A*
N4—H4A···N5^i^	0.86	2.23	2.945 (8)	140.
N6—H6···Cl1^ii^	0.86	2.46	3.266 (5)	156.

Symmetry codes: (i) *x*−1/2, −*y*+5/2, *z*−1/2; (ii) *x*+1/2, −*y*+3/2, *z*+1/2.
